# Microbial Diversity and Parasitic Load in Tropical Fish of Different Environmental Conditions

**DOI:** 10.1371/journal.pone.0151594

**Published:** 2016-03-28

**Authors:** Philipp Hennersdorf, Sonja Kleinertz, Stefan Theisen, Muslihudeen A. Abdul-Aziz, Grit Mrotzek, Harry W. Palm, Hans Peter Saluz

**Affiliations:** 1 Cell and Molecular Biology, Leibniz-Institute for Natural Product Research and Infection Biology, Jena, Germany; 2 Friedrich-Schiller-University, Jena, Germany; 3 Faculty of Agricultural and Environmental Sciences, University of Rostock, Rostock, Germany; 4 Faculty of Veterinary Medicine, Udayana University, Kampus Bukit Jimbaran, Indonesia; 5 Australian Centre for Ancient DNA, University of Adelaide School of Biological Sciences, Adelaide, Australia; Yeungnam University, REPUBLIC OF KOREA

## Abstract

In this study we analysed fecal bacterial communities and parasites of three important Indonesian fish species, *Epinephelus fuscoguttatus*, *Epinephelus sexfasciatus* and *Atule mate*. We then compared the biodiversity of bacterial communities and parasites of these three fish species collected in highly polluted Jakarta Bay with those collected in less polluted Indonesian areas of Cilacap (*E. sexfasciatus*, *A. mate*) and Thousand Islands (*E. fuscoguttatus*). In addition, *E. fuscoguttatus* from net cages in an open water mariculture facility was compared with free living *E. fuscoguttatus* from its surroundings. Both core and shared microbiomes were investigated. Our results reveal that, while the core microbiomes of all three fish species were composed of fairly the same classes of bacteria, the proportions of these bacterial classes strongly varied. The microbial composition of phylogenetically distant fish species, i.e. *A. mate* and *E. sexfasciatus* from Jakarta Bay and Cilacap were more closely related than the microbial composition of more phylogentically closer species, i.e. *E. fuscoguttatus*, *E. sexfasciatus* from Jakarta Bay, Cilacap and Thousand Islands. In addition, we detected a weak negative correlation between the load of selected bacterial pathogens, i.e. *Vibrio* sp. and *Photobacterium* sp. and the number of endoparasites. In the case of *Flavobacterium* sp. the opposite was observed, i.e. a weak positive correlation. Of the three recorded pathogenic bacterial genera, *Vibrio* sp. was commonly found in *E. fuscoguttatus* from mariculture, and lessly in the vicinity of the net cages and rarely in the fishes from the heavily polluted waters from Jakarta Bay. *Flavobacterium* sp. showed higher counts in mariculture fish and *Photobacteria* sp. was the most prominent in fish inside and close to the net cages.

## Introduction

Indonesia’s population growth and rapid economic development has led to an increased production of wastewater, from industry, farming and households [1]. Furthermore, inadequately purified wastewater is regularly disposed into coastal waters, resulting in a negative influence on marine ecosystems and its inhabitants [2]. Other anthropogenic activities such as capture fisheries and aquaculture also affect benthic communities as well as local fish communities and their environment [1, 3–5]. The consequences of these factors on the microbiome of fish and the possible implications on fish health are yet unknown. Within the coral triangle, Indonesian marine biodiversity exceeds that of any other place on earth [6]. This unique diversity includes all kinds of aquatic organisms, including marine fish, their parasites and pathogens. Fish parasites have been recognized as important sentinel organisms that are able to detect changes in environmental conditions [7–9]. Their diversity in tropical Indonesian waters is high, resulting in more than 80 different fish parasite species that have been recorded from groupers (*Epinephelinae*) kept under mariculture conditions [10]. It has been noted that the number of wild fish parasites exceeds that of mariculture fish [11]. This contrasts the observation that viral and bacterial disease outbreaks occur more regularly in mariculture fish, however, without any evidence for e.g. vibrioses or other bacteria caused skin diseases on Indonesian wild fish. It can be assumed that the environmental conditions, parasite infections and viral or bacterial disease outbreaks are linked and influence each other. According to Brown *et al*. [12], diet-induced altered microbiota results in dysbiosis that may result in inflammatory diseases in humans and contribute to an inappropriate inflammatory response. The microbiome of fish has been recently studied, with common core microbiome detected for certain fish species [13, 14]. The microbiome of marine fish revealed a rich biodiversity that predictably reacts to changing intestinal conditions. Xia *et al*. [15] recorded 33 phyla, 66 classes, 130 orders and 278 families in the intestinal microbiome of Asian seabass (*Lates calcarifer*). They also reported *Proteobacteria* (48.8%), *Firmicutes* (15.3%) and *Bacteroidetes* (8.2%) as the three most abundant bacteria taxa. Under starvation, *Bacteroidetes* were found to be dramaticaly enriched, while *Betaproteobacteria* was significant depleted. A comparison of the microbiome of fish from different environmental conditions such as mariculture and free-living has not yet been studied. In addition, while detailed parasitological investigations on important fish species such as e.g. *Lates calcarifer* [16] and *Epinephelus* spp. [9–11, 17] has been done, possible effects of parasite infection on fish microbiome is still unknown. As a result, we have sampled three important perciform Indonesian food fish species, the migrating pelagic yellowtail scad *Atule (A.) mate*, family Carangidae, less mobile sixbar grouper *Epinephelus (E.) sexfasciatus*, family Serranidae, and brown-marbled grouper *Epinephelus (E.) fuscoguttatus*, family Serranidae, from different water bodies and regions of Java. The samples were obtained from Jakarta Bay in the North of Jakarta (*A. mate*, *E. sexfasciatus*), a booming coastal megacity in Indonesia with over nine million inhabitants. The thirteen rivers that flow through this area receive enormous amounts of untreated wastewater from households and industries and discharge these high pollutant loads into Jakarta Bay [18, 19]. Comparative samples, representing cleaner water bodies, were collected at Pulau Seribu (*E. fuscuguttatus*), a chain of islands located to the North of Jakarta Bay, consisting of 110 islands stretching 45 km North into the Java Sea, added to the Thousand Islands Marine National Park in 2002, and from coastal waters off Cilacap (*A. mate*, *E. sexfasciatus*), a city at the South coast of Central Java.

## Materials and Methods

### Sample collection and examination

A total of 12 brown-marbled groupers *Epinephelus fuscoguttatus* (Forsskål, 1775), six sixbar groupers *Epinephelus sexfasciatus* (Valenciennes, 1828) and six yellowtale scads *Atule mate* (Cuvier, 1833) were studied from i) Jakarta Bay fish markets (Pasar Ikan Pelelangan: 6°06′17.7′′S 106°46′31.5′′E), ii) the 50 km remote Thousand Islands (Pulau Seribu: 5°44′13.3′′S 106°36′31.0′′E) National Park (North Java) and iii) Penyu Bay fish markets (Tempat Pelelangan Ikan (TPI)—Pelabuhan Perikanan Samudera Cilacap, 7°43′25.0′′S 109°01′22.7′′E), Cilacap (South Java), Indonesia (Table 1). All samples were collected during the 2012 rainy season.

**Table 1:** Experimental overview.

sample	host species	sampling location	sampling site	Number of parasites	*Vibrio* sp.	*Flavobacterium*	*Photobacterium* sp.
am1	*A. mate*	Jakarta	free-living	85	1.73	0.001	4.50
am2	*A. mate*	Jakarta	free-living	56	0.07	0.000	0.18
am3	*A. mate*	Cilacap	free-living	0	0.15	0.000	0.54
am4	*A. mate*	Cilacap	free-living	3	0.17	0.000	0.62
es1	*E. sexfasciatus*	Jakarta	free-living	58	0.019	0.002	0.18
es2	*E. sexfasciatus*	Jakarta	free-living	56	0.17	0.002	0.37
es3	*E. sexfasciatus*	Jakarta	free-living	70	0.48	0.001	0.44
es4	*E. sexfasciatus*	Cilacap	free-living	127	0.39	0.000	0.39
es5	*E. sexfasciatus*	Cilacap	free-living	51	2.56	0.001	1.48
es6	*E. sexfasciatus*	Cilacap	free-living	114	0.07	0.001	0.16
ef1	*E. fuscoguttatus*	Thousand Islands	free-living	23	48.43	0.001	20.66
ef2	*E. fuscoguttatus*	Thousand Islands	free-living	7	10.91	0.001	72.72
ef3	*E. fuscoguttatus*	Thousand Islands	free-living	9	2.86	0.001	90.87
ef4	*E. fuscoguttatus*	Thousand Islands	free-living	48	0.68	0.007	84.72
ef5	*E. fuscoguttatus*	Thousand Islands	mariculture	3	53.24	0.306	1.47
ef6	*E. fuscoguttatus*	Thousand Islands	mariculture	4	25.71	0.000	53.18
ef7	*E. fuscoguttatus*	Thousand Islands	mariculture	44	23.13	0.001	57.29
ef8	*E. fuscoguttatus*	Thousand Islands	mariculture	14	0.48	0.006	66.29
ef9	*E. fuscoguttatus*	Thousand Islands	mariculture	5	77.54	0.011	3.93
ef10	*E. fuscoguttatus*	Thousand Islands	mariculture	25	23.37	0.000	46.39

Investigated samples are listed by host species, sampling location and sampling site. The name of each sample is also given and corresponding to the figures and text. Additional the number of parasites per sample and the measured bacterial content for three fish pathogenic bacteria are listed.

The fish species *A. mate* and *E. sexfasciatus* were obtained from local fishermen; *E. fuscoguttatus* originated from an open water mariculture facility (Nusa Karamba Aquaculture) respectively were caught in the direct surrounding of the net cages by fish traps or with a fishing rod. All fish purchased from the market were declared as fresh for human consumption. Fish from Thousand Islands were dissected in the local laboratory (Nusa Karamba Aquaculture) right after catching, purchased fishes were separated into plastic bags and transported immediately to the laboratory or kept on ice and then frozen (≈ −20°C) until subsequently dissected at the Faculty of Biology, Jenderal Soedirman University, Purwokerto (UNSOED) and the Faculty of Veterinary Medicine. Total fish length (TL), standard fish length (SL), total weight (TW), slaughter weight (SW) and liver weight (not shown, used for the calculation of the hepatosomatic index were measured to the nearest 0.1 cm and 0.1 g prior to the parasitological examination [20] (Table 2).

**Table 2:** Fish morphometrical data.

fish species	area	sample collection point	n	TL [cm]	SL [cm]	TW [g]	SW [g]	m	f	juvenile	HSI	K	H’ total	H’ endo	E total	E endo
*Epinephelus fuscoguttatus*	Thousand Islands	fish pond	7	24.90 (23.1–26.2)	21.25 (20.1–22.4)	296.98 (249.5–354.2)	260.20 (221.7–302.2)	5	-	2	1.4	1.685	1.123	0.195	0.689	0.282
*Epinephelus fuscoguttatus*	Thousand Islands	free-living	5	27.60 (18.5–36.4)	24.02 (16.3–32.0)	475.44 (115.7–955.0)	427.16 (101.1–843.7)	-	-	5	1.1	2.032	1.000	0.300	0.5	0.22
*Epinephelus sexfasciatus*	Cilacap	fish market	3	27.33 (26.7–28.2)	22.60 (22.0–23.4)	330.13 (299.9–369.2)	283.07 (185.9–358.0)	-	3	-	1.39	0.006	1.301	0.981	0.626	0.548
*Epinephelus sexfasciatus*	Jakarta	fish market	3	24.83 (24.2–25.2)	20.50 (19.7–21.1)	231.07 (208.3–256.0)	218.77 (198.6–238.6)	-	-	3	1.43	0.006	0.933	0.620	0.521	0.386
*Atule mate*	Cilacap	fish market	3	24.20 (22.2–25.6)	19.50 (18.1–20.7)	150.00 (114.3–180.3)	140.07 (107.7–168.1)	2	-	1	1.4	0.988	0.451	0.451	0.650	0.650
*Atule mate*	Jakarta	fish market	3	26.20 (25.3–27.4)	21.20 (20.4–22.5)	177.20 (166.7–188.9)	165.27 (155.9–178.5)	3	-	-	0.8	0.919	1.436	1.118	0.578	0.54

total (TL) and standard length (SL) in cm, total (TW) and slaughter weight (SW) in g, hepatosomatic index (HSI) and condition factor (K) from different Indonesian sampling sites during rainy season 2012. Additionally given are the Shannon-Wiener diversity index and Evenness for all parasites in a sample (H’ total; E total) and calculated only for the endoparasites (H’ endo; E endo), m: male, f: female

Parasitological examination followed Palm & Bray [21]. Skin, fins, eyes, gills, nostrils, mouth- and gill cavity were examined for ectoparasites. Inner organs such as the digestive tract, liver, gall bladder, spleen, kidneys, gonads, heart and swim bladder were separated and transferred into saline solution for microscopically examination under the stereomicroscope (Zeiss Stemi DV4) in order to allow a quantitative parasitological examination of each organ; belly flaps and musculature (fillets) were examined on a candling table. Isolated parasites were fixed in 4% borax-buffered formalin and preserved in 70% ethanol. Finally, the musculature was sliced into 0.5–1 cm thick filets, pressed between two petri dishes to identify and isolate parasites from the musculature. Nematoda were dehydrated in a graduated ethanol series and transferred to 100% glycerine (Riemann, 1988). Digeneans, monogeneans and cestodes were stained with acetic carmine, dehydrated, cleared with eugenol and mounted in Canada balsam, whereas crustaceans were dehydrated and transferred directly into balsam. The identification of parasites was based on original descriptions [22–39].

During parasitological investigation feces samples were collected. The intestine was carefully cut and feces (without bones or big, solid components) was scraped with a scoop and stored in 99.9% EtOH for subsequent analyses at the Leibniz Institute for Natural Product Research and Infection Biology e.V. Hans-Knöll-Institute (HKI), Jena, Germany.

### Parasitological parameters

Parasitological calculations followed Bush *et al*.[40] The present study applies the method by Palm *et al*.[7], Palm & Rueckert [9], Kleinertz & Palm [41] and Kleinertz *et al*. [42] to monitor the parasite community of Indonesian fish. This is based on the assumption that data and parameters based on the prevalence of certain parasites are characteristic for undisturbed environmental conditions with high parasite diversity. Ecological parameters were evaluated to indicate regional differences, such as the diversity indices Shannon-Wiener and Evenness (both for all parasites as H’ total resp. E total, and for the endoparasites exclusively as H’ endo resp. E endo, see Kleinertz & Palm [41] and Kleinertz *et al*. [42]), fish ecological indices such as the hepatosomatic index, condition factor and parasitological parameters such as ecto- endoparasite ratio and differences in prevalence of metazoan parasite infections [7, 9, 42].

The total diversity (Shannon–Wiener diversity index [43] H’) and the Evenness index (E) of Pielou [44] were calculated for each fish species. According to Kleinertz *et al*. [41] the hepatosomatic index was calculated as a descriptor of a possible pollution impact to the fish host, which may affect increasing liver weights (LW) in relation to the total weight (TW) of the host [45].

### Isolation of microbial genomic DNA

Feces samples (5 to 10 mg per specimen) were homogenized in lysis buffer (Bio u. Sell) using a Precellys tissue homogenizer with 1.4 mm ceramic beads and a 3× 30 s homogenization time with 30 s pause at 5000 rpm. Samples were incubated for one hour at 37°C. Subsequently, RNaseA was added and incubation was done for one hour at 37°C and subjected to an over-night proteinase K digestion. Whole genomic DNA was extracted using a Phenol/Chloroform/Isoamyl alcohol extraction followed by ethanol precipitation and quantification.

### PCR amplification and Sequencing

Universal prokaryotic primers F515/R806 was used to amplify the V4 region of the bacterial/archaeal 16S rRNA gene [46, 47]. Primers were modified to include Illumina Nextera flowcell adapter sequences, additional forward and reverse primer pads to avoid primer-dimer formation, and a 2-bp linker sequence not matching against any 16S rRNA sequence immediately upstream of the gene primer [47]. The reverse primers also incorporated 12-bp error-correcting Golay barcodes [47].

For each individual sample, three 20 *μ*l PCR reactions (and a negative control) were set up containing 10 ng genomic DNA, 1.25 U TaKaRa SpeedSTAR HS DNA polymerase, 0.2 *μ*M of each primer, corresponding Fast Buffer 1, and 200 *μ*M dNTP final concentration. Reactions were performed with an initial denaturation step for 3 min at 95°C followed by a 40-cycle amplification (95°C for 10 s, 62°C for 30 s), and a final elongation step of 2 min at 72°C on an Applied Biosystems 9800 Fast Thermal Cycler. PCR products were visualized using gel electrophoresis and for successful samples blue, replicate reactions were combined and primer multimers, polymerase, and dNTPs were removed using the Agencourt AMPure XP post-PCR cleanup kit (Beckman Coulter).

Cleaned PCR-products were quantified using the Agilent 2100 Bioanalyser, pooled in equimolar concentration, and subjected to 250-bp paired-end amplicon sequencing on an Illumina MiSeq platform at StarSEQ GmbH (Mainz). Raw sequence data are deposited at NCBI’s Short Read Archive under accession number SRP059667.

### Ethics statement

In this study, experiments were not performed on live vertebrates. Instead, freshly caught dead fish was used and therefore no ethics statement is required. Samples were taken within the INDONESIAN GERMAN JOINT RESEARCH COOPERATION “Science for the protection of Indonesian marine Coastal Ecosystems—A GERMAN INDONESIAN INITIATIVE IN EARTH SYSTEM RESEARCH. With research permit from RISTEK, the INDONESIAN STATE Ministry of research and technology.

### Data processing and statistical analyses

Raw sequence base call files (bcl) were converted into FASTQ format and demultiplexed using the CASAVA v1.8.2 (Illumina) software. Clustering of the reads into Operational Taxonomic Units (OTUs) was performed using the uparse pipeline as implemented in usearch 7.0.1090 [48]. Before clustering, a number of preprocessing steps were carried out: Paired reads were merged using the fasta_mergepairs command with a minimum Phred score cutoff threshold of 5 and a minimum overlap length of 75 bp. Merged reads were trimmed to a length of 250 bp and filtered if the expected number of errors exceeded 0.5 (fastq_filter). Filtered reads were pooled across samples and dereplicated using the derep_fulllength command. The dereplicated reads were sorted by abundance and all singletons were discarded.

The resulting high-quality sequences were grouped into OTUs using the UPARSE-OTU algorithm [48] (cluster_otus) at a 97% sequence similarity cutoff. This step includes chimera filtering based on models built from more abundant reads. An additional reference-based chimera filtering step was performed using the UCHIME algorithm [49] (uchime_ref) and the ChimeraSlayer reference database (http://microbiomeutil.sourceforge.net). The remaining sequences were considered OTU representative sequences or phylotypes, and mapped against the filtered sample reads at an identity threshold of 97% (usearch_global) to create an OTU abundance table.

OTU sequences were assigned to a taxonomic lineage by inferring the lowest common ancestor for the top BLAST matches against the Greengenes database [50]. Only BLAST hits with a query coverage above 75% and a bitscore above a cutoff value of 97% of the bitscore achieved by the best hit, were considered. Community analyses were performed in R [51] using the packages phyloseq [52] and DESeq2 [53], as well as vegan [54] for diversity analysis.

## Results

### Analysis of fecal bacterial communities

In total, 8,453,888 valid sequence reads binned into 484 Operational taxonomic units (OTU) were retrieved from 24 fecal samples of the three target species. Three samples (am5, am6 and ef12) were excluded from further analyses due to low number of reads (180, 17,022, and 519 reads, respectively). In addition, one sample from *E. fuscoguttatus* (ef11) was filtered out, due to stochastic behavior of the microbial community, caused by sampling problems. The remaining 20 samples yielded on average 413,100 mapped reads ranging from 154,600 to 719,100 reads. Two OTUs with 1,938 and 40 reads, respectively, were of mitochondrial and chloroplast origin and excluded from subsequent analyses. The remaining 482 OTUs were classified into a total of 19 different phyla, in decreasing order of abundance: *Proteobacteria* (85.93%), *Firmicutes* (11.47%), *Fusobacteria* (1.84%), *Spirochaetes* (0.48%), *Actinobacteria* (0.11%), *Bacteroidetes* (0.06%), *Acidobacteria* (0.04%), *Chlamydiae* (0.02%), *Lentisphaerae* (0.02%), *Cyanobacteria* (0.01%), *Verrucomicrobia*, *Planctomycetes*, *Armatimonadetes*, *WPS-2*, *Tenericutes*, *Chloroflexi*, *Nitrospirae*, *TM6*, *Thermi* (<0.01%).

**Fig 1:**
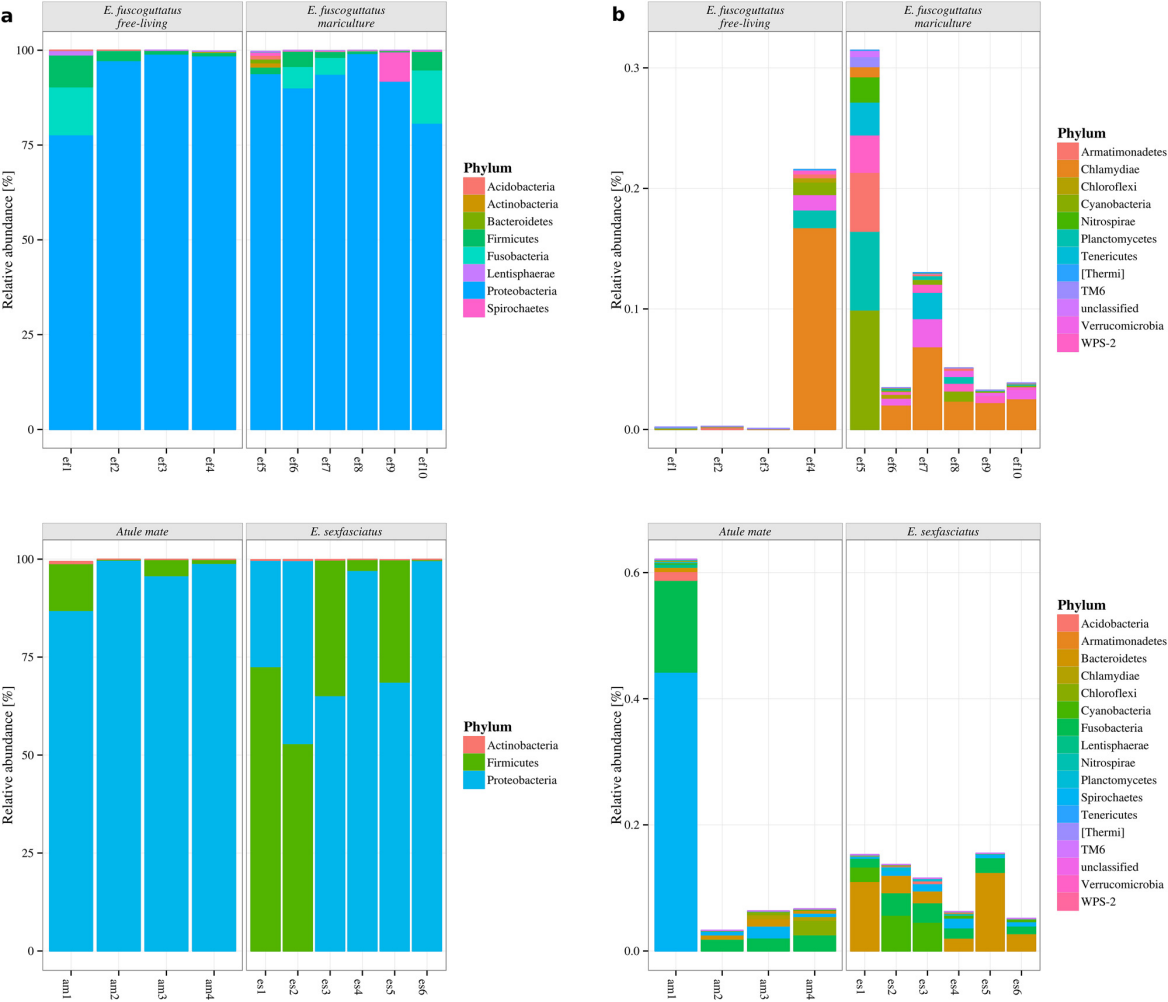
Taxonomic summary of predominant (a) and rare (b) phyla across the three fish species. To determine the predominant phyla only OTUs with an abundance of over 0.01% per sample were selected, resulting in three phyla for *A. mate* and *E. sexfasciatus* and eight phyla for *E. fuscoguttatus*. To determine rare phyla with relative abundance counts of less than 0.01% are included in this plot. ef1-ef10 refers to all samples from *E. fuscoguttatus*, while am1-am4 belongs to *A. mate* and es1-es6 to *E. sexfasciatus*.

For all three fish species, the predominant bacterial phyla were *Proteobacteria* (average (avg) 85.39%, standard deviation (s.d.) 19.73%) followed by *Firmicutes* (avg 11.88%, s.d. 20.15%) and *Actinobacteria* (avg 0.13%, s.d. 0.26%) (Fig 1a). Considering only bacterial phyla with a relative abundance of more than 0.1%, *E. fuscoguttatus* showed a markedly higher bacterial diversity than both *E. sexfasciatus* and *A. mate* (eight vs. three phyla).

Across the rare phyla, *A. mate* and *E. sexfasciatus* had an increased bacterial diversity with 16 phyla against 11 phyla for *E. fuscoguttatus*, notably the five additional phyla appeared predominantly in *E. fuscoguttatus* samples. Among the remaining 11 phyla, three different host species had a different composition. Three of the *A. mate* samples, two from Cilacap (am3, am4) and one from Jakarta (am2), showed low abundances, whereas one sample from Jakarta (am1) had a higher abundance of *Spirochaetes* (0.44%) and *Fusobacteria* (0.14%). The samples derived from *E. sexfasciatus* shared in general a similar composition of rare bacterial phyla, consisting mainly of *Bacteriodetes* (0.06%), *Fusobacteria* (0.02%) and *Cyanobacteria* (0.02%). At least one sample of *E. fuscoguttatus*, from outside (ef4) the net cages offered a higher abundance for *Chlamydiae* (0.17%), meanwhile the other samples had nearly none abundance for rare phyla (Fig 1b).

**Fig 2:**
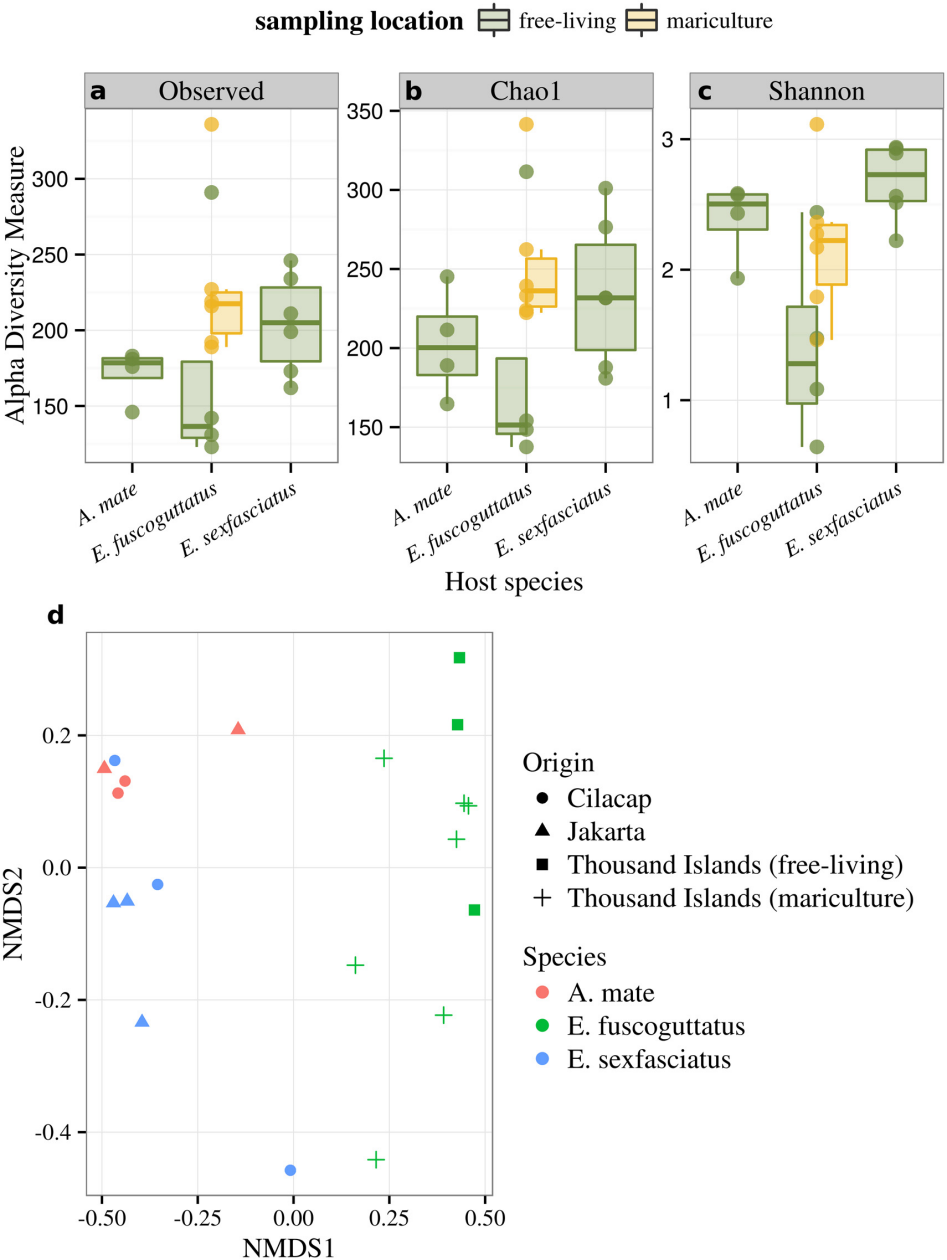
Alpha diversity and beta diversity estimates across fish species, sampling location and visualization of differences in bacterial gut community composition by host species. **a**: observed OTU richness; **b**: Chao1 index that estimates the true species richness of a sample; **c**: Shannon-Wiener diversity index accounting for species abundance and eveness of distribution. Dots represent estimates for individual samples, solid lines constitute the median, boxes the quartiles, and bars the interquartile range. **d**: Beta diversity is estimated with Nonmetric multidimensional scaling (NMDS) of bacterial communities derived from 20 fish specimen coloured by host species. Point shapes indicates differences in the sampling location. Samples derived from mariculture are labelled accordingly. Ordinations are based on between-sample dissimilarities calculated by Bray-Curtis distances.

In order to assess diversity of the microbial communities, all samples were rarefied to same library size, resulting in 90 OTUs that were sorted out. Following which, three statistical models were applied. The observed OTU richness (Fig 2a) of *A. mate* showed three samples with a related number of OTUs (am1, am3, am4) and one sample from Jakarta (am2) with a reduced number of OTUs, resulting in a median of 178 OTUs. For *E. sexfasciatus*, the median observed OTU richness was 205, distributed to three samples below (es1, es5, es6) and three samples above (es2, es3, es4) the median value. The samples of *E. fuscoguttatus* displayed a higher variation. Free-living samples offered the lowest median observed OTU richness with 136 OTUs, whereas deviation between the samples was very high. In contrast samples derived from mariculture showed a higher observed OTU richness with a median value of 217 OTUs. The nonparametric richness estimator Chao1, providing a statistical estimation of the true species richness of a community including unobserved species [55], revealed a high difference between the observed and the expected OTU richness in the samples of *A. mate* (200 OTUs), *E. sexfasciatus* (231 OTUs) and *E. fuscoguttatus* from mariculture (236 OTUs). Only free-living samples had a small difference between expected and observed OTUs (151 OTUs) (Fig 2b). This implies that an even greater diversity of bacteria lies undiscovered. The ecological diversity, measured by Shannon-Wiener diversity index, revealed high bacterial diversity in the samples of *A. mate* (2.5) and *E. sexfasciatus* (2.7). In addition, the mariculture samples from *E. fuscoguttatus* displayed higher bacterial diversity (2.2) than the free-living samples (1.3) (Fig 2c). To measure the differences between the bacterial gut community compositions a nonmetric multidimensional scaling method was used to obtain ordinations based on between-sample dissimilarities calculated by Bray-Curtis distances [56]. The ordinations displayed two different clusters, whereas two samples (am1, es5) were outliers. One cluster was formed by *A. mate* and *E. sexfasciatus*. This implied a closer relationship of the microbial communities of *A. mate* and *E. sexfasciatus* than the communities of *E. fuscoguttatus*, which formed the other cluster (Fig 2d). Both fish species were collected from off Cilacap and inside Jakarta Bay, while *E. fuscoguttatus* originated from the Thousand Islands. This relationship stands in contrast to the phylogeny of the host species, whereas *E. fuscoguttatus* and *E. sexfasciatus* belonging to the same genus must be more closely related than to *A. mate* related at order level. Further analysis, using the statistical method adonis, confirmed the significance of these clusters with an p-value below 0.001.

### Comparison between the microbiomes of free-living and mariculture *E. fuscoguttatus*

Comparing the fecal bacterial communities of *E. fuscoguttatus* over different sampling sites, inside the net cages and outside on the surrounding reef, revealed only minor differences in the composition of most abundant phyla. Proportions of *Proteobacteria* (avg free-living (free): 93.14% vs. avg mariculture (mari): 91.58%, s.d. free: 10.30% vs. s.d. mari: 6.10%) and *Spirochaetes* (avg free: 0.01% vs. avg mari: 1.52%, s.d. free: 0.02% vs. s.d. mari: 3.06%) differed slightly between free-living samples and samples from mariculture. Also minor differences in the proportion of *Fusobacteria* could be detected (avg free: 3.18% vs. avg mari: 4.06%, s.d. free: 6.30% vs. s.d. mari: 5.46%). The other dominating phyla appeared nearly in same ratios (Fig 1a).

Among the low abundance phyla no difference was detected based on differences in sampling sites. In each case one sample from inside (ef5) and outside the net cages (ef4) displayed a higher abundance of detected phyla, but this could not be assigned to different sampling sites. (Fig 1b). Differences between the bacterial gut community compositions of *E. fuscoguttatus* specimen showed that the samples from inside the net cages formed a subcluster within the cluster of *E. fuscoguttatus* (Fig 2d). Smaller distances between samples from inside pointed out a more conserved community structure in comparison to the samples from outside the net cages. Median observed OTU richness (Fig 2a) revealed a reduced OTU richness for the free-living samples of *E. fuscoguttatus* with 136 OTU. In contrast samples derived from inside the net cages showed a higher observed OTU richness with 217 OTUs, supported by Chao1 richness estimator showing a predicted number of 236 OTUs for samples from inside in contrast to 151 OTUs for the samples from outside (Fig 2b). In addition, Shannon-Wiener diversity index indicated a more diverse bacterial community structure for inside (2.22) than the free-living specimens (1.28) (Fig 2c).

### Core and shared microbiomes

**Fig 3:**
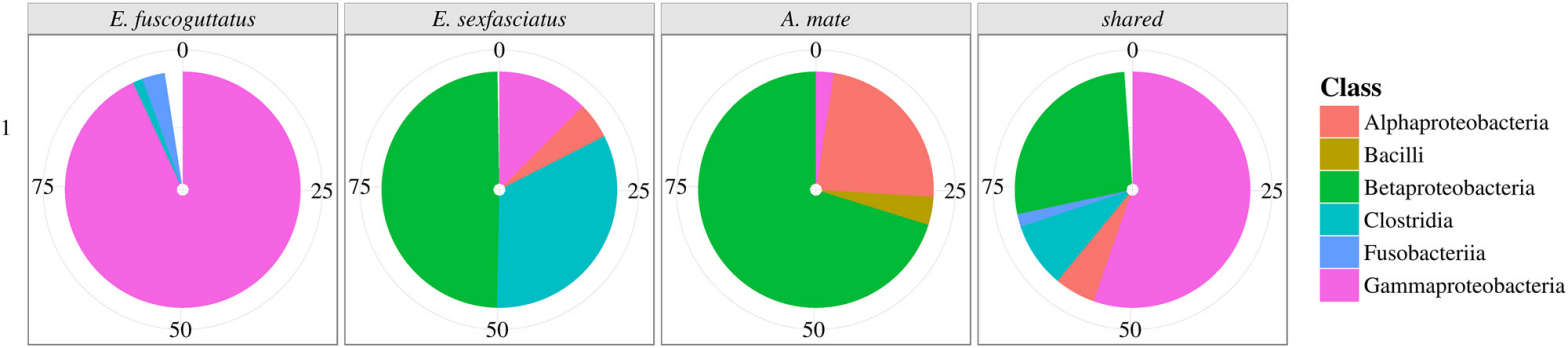
Core microbiome for each host species and shared microbiome on class level. The core microbiome is constructed by counting OTUs that are present in every sample of each host species. The shared microbiome results as a combination of all three core microbiomes, representing all OTUs in all samples of the three host fish species.

The comparison of shared OTUs revealed a different core microbiome for each host species (Fig 3). Core microbiome construction lead to a high number of shared OTUs for each of the three host species (*E. fuscoguttatus*: 106 OTUs, *E. sexfasciatus*: 129 OTUs and *A. mate*: 124 OTUs). Core microbiome of *E. fuscoguttatus*, consisting of 106 OTUs, was dominated by *Gammaproteobacteria* with over 92.59% whilst *Fusobacteria* (3.12%), *Clostridia* (1.35%) and *Betaproteobacteria* (1.00%) constituted the rest of the core microbiome. *E. sexfasciatus* revealed a completely different composition with 129 OTUs belonging to the core microbiome. The core was dominated by *Betaproteobacteria* with a relative abundance of 49.16%. Also a huge portion of *Clostridia* (32.67%) and a lower portion of *Gammaproteobacteria* (12.45%) and *Alphaproteobacteria* (4.97%) were revealed. Core microbiome of *A. mate* consisted of 124 OTUs, and was also dominated by *Betaproteobacteria* (69.84%) and had a large portion of *Alphaproteobacteria* (23.40%). The rest of it was distributed to *Bacilli* (3.82%) and *Gammaproteobacteria* (2.43%). Out of the three different core microbiomes a shared microbiome was constructed, by counting only OTUs present in every sample of the three host species. Thereby the resulted shared microbiome was dominated by *Gammaproteobacteria* (55.08%) and *Betaproteobacteria* (27.07%). *Clostridia* (8.91%), *Alphaproteobacteria* (5.57%), *Fusobacteria* (1.76%), as well as *Bacilli* (0.93%) and *Brevinematae* (0.50%) formed the rest of this shared microbiome.

### Parasites

Fish parasitological studies on *E. fuscoguttatus*, *E. sexfasciatus* and *A. mate* from Thousand Islands (Pulau Seribu), Cilacap and Jakarta (Table 1), revealed 28 different parasite species belonging to the following taxa: 10 Digenea, 4 Monogenea, 1 Cestoda, 7 Nematoda, 2 Acanthocephala, 1 Hirudinea and 3 Crustacea (Table 3). In an additional study, using a shotgun sequencing approach on *Epinephelus fuscoguttatus*, all observed parasites were confirmed [57]. Data on prevalence, intensity, mean intensity and mean abundance of the collected parasite species for each fish species are summarized in Table 3. Parasite species richness of up to 12 taxa, calculated and pooled in the fish samples for both sites (Jakarta Bay and Cilacap) was highest in *A. mate* followed by *E. sexfasciatus* with nine taxa. *E. fuscoguttatus* from both sampling locations in the Thousand Islands had only seven taxa, with only five species from the fish in the net cages and seven from the fish caught in the reef beside the net cages.

**Table 3:** Parasitic load.

		*Atule mate* Jakarta				*Atule mate* Cilacap				*E. fuscoguttatus* free PS				*E. fuscoguttatus* caged PS				*E. sexfasciatus* Cilacap				*E. sexfasciatus* Jakarta			
parasite taxa	site	P[%]	MI	(I)	MA	P[%]	MI	(I)	MA	P[%]	MI	(I)	MA	P[%]	MI	(I)	MA	P[%]	MI	(I)	MA	P[%]	MI	(I)	MA
***Didymodiclinus*** **sp. (D)**	**fin, skin**																	100.0	5.7	(5–6)	5.7				
***Echinostoma*** **sp. (D)**	**in., st**.	33.3	1.0	(1)	0.3																				
***Genolinea*** **sp. (D)**	**st**.	66.7	7.5	(3–12)	5.0																				
***Prosorhynchus cf. australis*** **(D)**	**in., pyl**.									20.0	2.0	(2)	0.4												
***Prosorhynchus crucibulum*** **(D)**	**in**.									40.0	1.0	(1)	0.4												
***Prosorhynchus*** **sp. (D)**	**in., pyl., st**.																	100.0	29.3	(9–55)	29.3	66.7	4.5	(2–7)	3.0
***Pseudopecoelus*** **sp. (D)**	**mes. of swb**.									20.0	235.0	(33–437)	94.0	14.3	1.0	(1)	0.1								
***Didymozoidae indet***. **(D)**	**in., st**.	66.7	44.0	(5–66)	29.3	33.3	1.0	(1)	0.3																
**Digenea indet. I (D)**	**st**.	66.7	2.5	(2–3)	1.7																				
**Digenea indet. II (D)**	**in**.	33.3	1.0	(1)	0.3																				
***Haliotrema cf. epinepheli*** **(M)**	**gi., gicv**.																	100.0	14.7	(9–25)	14.7	100.0	36.0	(27–41)	36.0
***Pseudorhabdosynochus epinepheli*** **(M)**	**gi**.									60.0	3.0	(1–6)	1.8	85.7	9.5	(1–22)	8.1								
***Pseudorhabdosynochus lantauensis*** **(M)**	**gi., gicv., op**.									100.0	13.4	(2–34)	13.4	85.7	6.8	(1–19)	5.9								
**Mazocraeidea indet. (M)**	**gi**.	33.3	1.0	(1)	0.3																				
***Callitetrarhynchus gracilis*** **(C)**	**bcv., mes., gicv., go., li., stw**.	100.0	7.0	(4–9)	7.0																				
***Camallanus*** **sp. I (N)**	**in**.	66.7	1.0	(1)	0.7																				
***Camallanus*** **sp. II (N)**	**pyl**.																	33.3	1.0	(1)	0.3	33.3	1.0	(1)	0.3
***Capillaria*** **sp. (N)**	**gi**.																	33.3	1.0	(1)	0.3	66.7	1.0	(1)	0.7
***Hysterothylacium*** **sp. I (N)**	**in., st**.	66.7	2.0	(1–3)	1.3																				
***Hysterothylacium*** **sp. II (N)**	**bcv. (mes.), go., li., stw**.																	100.0	45.0	(11–101)	45.0	100.0	21.0	(12–35)	21.0
***Philometra*** **sp. (N)**	**bcv., go., li**.																	100.0	1.3	(1–2)	1.3				
***Raphidascaris*** **sp. (N)**	**bcv. in fat; stw**.									40.0	1.0	(1)	0.4	28.6	3.0	(1–5)	0.9								
***Rhadinorhynchus lintoni*** **(A)**	**in**.				66.7	2.5	(2–3)	1.7																	
***Serrasentis sagittifer*** **(A)**	**mes. of go., in., st**.																					33.3	1.0	(1)	0.3
***Zeylanicobdella arugamensis*** **(H)**	**gicv., fin**									60.0	1.6	(3–4)	2.7	85.7	1.7	(1–3)	1.4								
***Caligus*** **sp. (C)**	**mcv**.	100.0	2.7	(1–4)	2.7																				
***Lepeophtheirus*** **sp. (C)**	**gi**.																	33.3	2.0	(2)	0.7				
**Bomolochidae indet. (C)**	**gi**.	33.3	2.0	(2)	0.7																				
	**ectoparasites**	3				0				3				3				2				1			
	**endoparasites**	8				2				4				2				6				5			
	**Ec/En ratio**	0.4				0.0				0.8				1.5				0.3				0.2			

The prevalence [%], intensity (I), mean intensity (MI) and mean abundance (MA) of ectoparasites and endoparasites from *E. fuscoguttatus*, *E. sexfasciatus* and *A. mate* from different Indonesian waters. Additionally given is the amount of ecto- and endoparasite species as well as the Ec/En ratio. bcv: body cavity, gi: gills, gicv: gill cavity, go: gonads, in: intestine, li: liver, mes: mesenteries, mcv: mouth cavity, pyl: pylorus, st: stomach, stw: stomach wall; A: Acanthocephala, C: Cestoda, Cr: Crustacea, D: Digenea, H: Hirudinea, M: Monogenea, N: Nematoda

To analyze parasite composition at each sampling site, the Shannon-Wiener diversity index suggested as an ecological parameter by Palm *et al*. [7] and Palm & Rueckert [9] were calculated (Table 2). Highest total Shannon-Wiener diversity was given in *A. mate* from Jakarta (1.44) followed by *E. sexfasciatus* from Cilacap (1.30) while lowest total diversity was observed in *A. mate* from Cilacap (0.45). Highest endoparasite Shannon-Wiener diversity was seen in *A. mate* in Jakarta (1.12), medium in *E. sexfasciatus* from both samples (0.98 vs. 0.62) and low in *A. mate* from Cilacap (0.45), *E. fuscoguttatus* from the surroundings of the net cages (0.30) respectively inside the net cages (0.20) (Table 2). Ecto-/endoparasite ratios calculated by using the number of ectoparasite species vs. the number of endoparasite species ranged from 0.0 (*A. mate*, Cilacap) up to 1.5 (*E. fuscoguttatus*, Thousand Islands from net cages) (Table 2). Additional ecological parameters, such as the hepatosomatic index and condition factors were calculated (Table 2) and showed highest HSI values in *E. fuscoguttatus* and *A. mate* (cultured at Thousand Islands respectively Cilacap, both = 1.4). All other HSI values ranged between 0.6 and 1.1. The condition factor was highest in both samples from *E. fuscoguttatus* from Thousand Islands (1.69–2.03).

### Correlation between pathogenic bacteria and parasites

**Fig 4:**
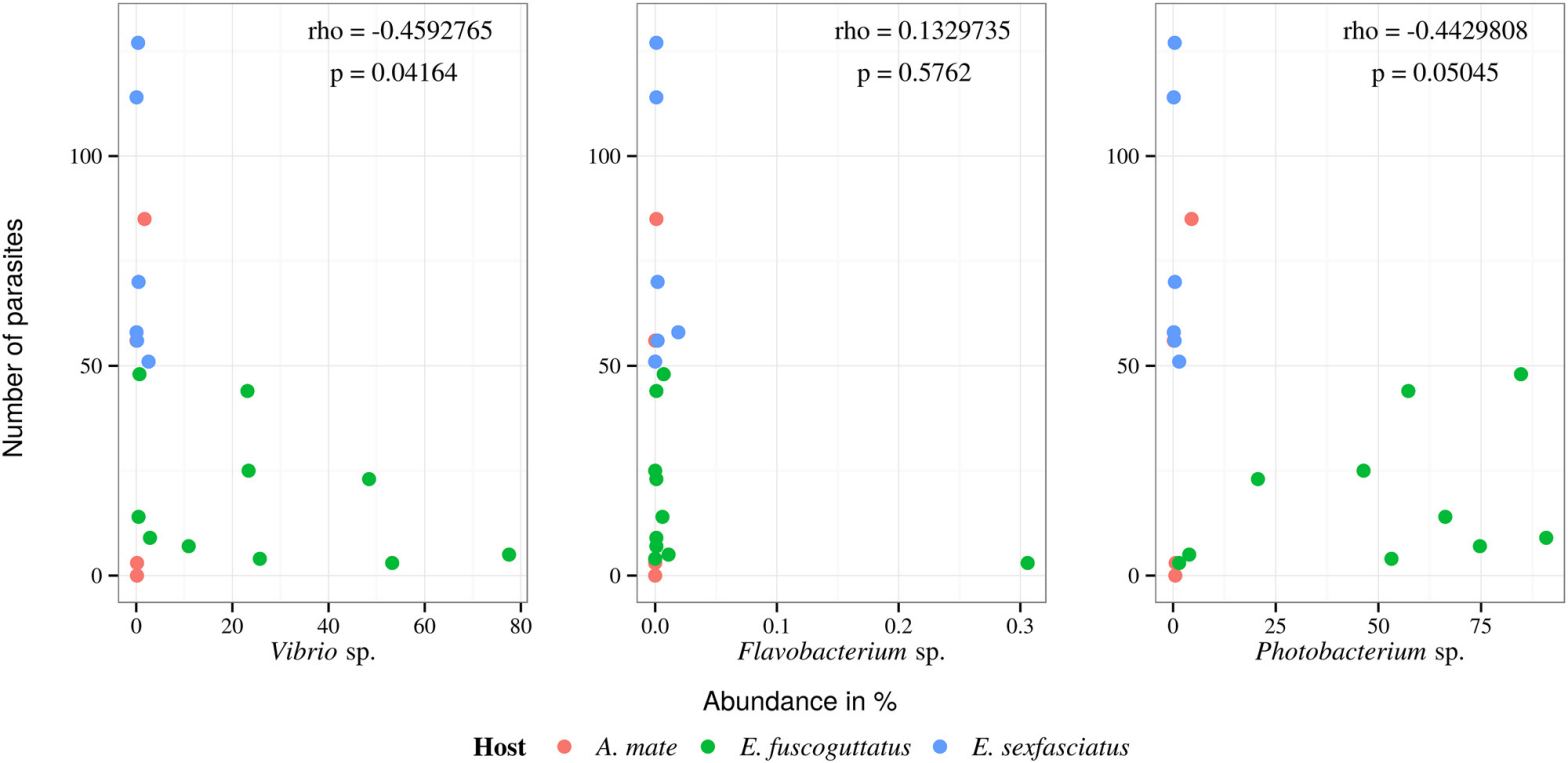
Spearman’s rank-order correlation was performed to determine a relationship between the numbers of parasites against the abundance of three known fish pathogenic bacteria. It showed for *Vibrio* sp. (*ρ* = −0.4592765, *p* = 0.04164) and *Photobacterium* sp. (*ρ* − 0.04429808, *p* = 0.05045) a medium negative correlation and a weak positive correlation for *Flavobacterium* sp. (*ρ* = 0.1329735, *p* = 0.5762).

A Spearman’s rank-order correlation between commonly known fish pathogenic bacteria, selected from microbiome analysis and recorded parasite number, in part also reflect the observed biodiversity (Fig 4). The highest parasite numbers were observed for free-living *E. sexfasciatus* from Cilacap (es4, es6), followed by *A. mate* and *E. sexfasciatus* from Jakarta Bay, mariculture and free-living *E. fuscoguttatus* from Thousand Islands. *A. mate* from Cilacap displayed virtually no parasite infection. All fish with a high number of parasites (above 50 individual metazoans) had no potentially pathogenic *Vibrio* sp., *Flavobacterium* sp. or *Photobacterium* sp. This was supported by the results of a Spearman’s rank-order correlation test, which revealed a medium negative correlation for *Vibrio* sp. (*ρ* = −0.4592765, *p* = 0.04164) and *Photobacterium* sp. (*ρ* = −0.4429808, *p* = 0.05045). On the other hand, the highest *Vibrio* sp. counts were found in *E. fuscoguttatus* from inside the net cages (ef10, ef5) and, to a much lower degree, in *E. fuscoguttatus* outside the net cages (ef1) from the surrounding reef. An increased detected value for *Flavobacterium* sp. was only recorded from a fish inside the net cages without metazoan parasites (ef5), resulting in a weak positive correlation (*ρ* = 0.1329735, *p* = 0.05762) with a high p-value. *Photobacterium* sp. could only be recorded from *E. fuscoguttatus*, from free-living and mariculture fish from Thousand Islands, without any record from Jakarta Bay and off Cilacap.

## Discussion

Our investigation of microbial composition reveals three phyla that are predominantly present across all three host species: *Proteobacteria*, *Firmicutes* and *Actinobacteria*. Particularly, *Proteobacteria* immensely dominates on phyla level in all samples, composed of *Gammaproteobacteria*, *Betaproteobacteria* and *Alphaproteobacteria* on the class level. In addition, *Firmicutes* and *Actinobacteria* was detected in all samples. In line with other metagenomic studies of fish microbiomes, these three phyla have been recognized as the characterstic components of the fish microbiome [16, 58]. For example, Asian sea bass has, *Proteobacteria* (48.8%), *Firmicutes* (15.3%), *Bacteroidetes* (8.2%) and *Fusobacteria* (7.3%) as the four most abundant bacterial phyla [15]. While members of the predominant bacterial communities at phylum level appears equal over all three host species and similar to earlier studied fish, the proportion of these communities is unique for every host species. This assumption is supported by beta diversity analysis (Fig 2d), which presents a distinct cluster for each of the three host species. Investigations on rare phyla (below 0.1%) results in altered phyla and proportions for every sample [59], with no detectable difference on sampling locations or host species.

Comparing samples derived from *E. fuscoguttatus* under mariculture and free-living conditions, we detected similar compositions of predominant bacterial communities. Further investigation of differences in bacterial community composition revealed that samples from mariculture formed a subcluster within free-living samples, indicating robustness of the bacterial communities within these samples compared to communities in free-living samples. In contrast, alpha diversity measurements exposed higher species richness for the mariculture samples, furthermore mean number of the expected species richness was higher compared to free-living samples. This indicated that the number and distribution of phyla within both conditions were the same, but deeper taxonomical levels revealed a more diverse bacterial community for the mariculture samples in contrast to the free-living ones.

The exploration of the core microbiomes resulted in three cores with similar members under different proportions. While the core microbiomes of *A. mate* and *E. sexfasciatus* consisted of a dominating parts of *Betaproteobacteria*, the core of *E. fuscoguttatus* is highly dominated by *Gammaproteobacteria* with over 90%, also a member in the other core microbiomes, but only with a portion of 12% (*E. sexfasciatus*) and 3% (*A. mate*). In addition, *Clostridia* was proportionally higher in *E. sexfasciatus*, but had negligible presence in *E. fuscoguttatus* and *A. mate*. This was also observed with *Alphaproteobacteria*, present in large proportions in *A. mate* compared to, *E. sexfasciatus* and none on *E. fuscoguttatus*. Three bacteria were only detected in one of the core microbiomes: *Fusobacteria*, *Brevinematae* in *E. fuscoguttatus* and *Bacilli* in *A. mate*. The shared microbiome derived by combining the three core microbiomes from the host species, resulted in the composition of all three core microbiomes, whereas *Gammaproteobacteria* (54.72%) and *Betaproteobacteria* (26.59%) dominated. These three different core microbiomes are supported by calculations from beta diversity analysis, showing three separate clusters, each consisting only of one host species including all environmental conditions (Fig 2d). Furthermore, core microbiomes showed that bacterial communities differ with host species. With a unique bacterial community per host species, the core microbiome is also unique.

Previous fish parasitological studies in Indonesian waters have revealed a rich species diversity, naming nearly 80 different taxa from mariculture groupers alone, belonging to the three genera *Epinephelus*, *Cromileptes* and *Plectropomus*. For cultured epinephelids in total 60 different parasite species were found. The highest parasite diversity was recorded for *E. fuscoguttatus* with 46 parasite species/taxa, 25 of which were ectoparasites and 21 were endoparasites. Another frequently cultivated fish, *Epinephelus coioides*, harbours 36 parasite species/taxa (21 ecto- and 15 endoparasites). While the lowest parasite diversity was found for *Epinephelus areolatus* (three ectoparasites only) [10]. Independent data from *E. sexfasciatus* and *A. mate* from Indonesian waters so far are unavailable.

In this study, we record seven different parasite species for *E. fuscoguttatus* from Thousand Islands, five from within and seven from outside the net cages, comparatively less than reported from the same location in earlier studies [17]. In general, wild fish has been observed to be infected by fish parasites more than cultured fish [10, 11]. Palm *et al*.[7] used fish parasites to monitor long-term change in finfish grouper mariculture in Indonesia. A total of 210 *Epinephelus fuscoguttatus* were sampled in six consecutive years between 2003/04 and 2008/09 from the same mariculture facility and, using the same methodology, examined for parasites. While fish from inside the net cages in the first dataset had 14–16 different parasite species, this number decreased to eight in the rainy season 2008/09. Palm *et al*.[7] stated that the diminishing parasite richness over time may reflect changing environmental conditions at the site, from the initiation of mariculture activity (beginning of the parasite monitoring) until increased fish production six years later. However, the authors sampled only fish from net cages. In the present study, only five parasite species occurred inside the fish from the cages, reflecting a further decrease in parasite richness in rainy season 2012. Our data demonstrates that parasite richness at the present time is even further reduced. More importantly, fish from outside the net cages had only seven different parasite species. This fact strongly supports the notion that not only the feed within the mariculture facility but also environmental conditions must have changed during the last and present investigation.

Rueckert *et al*.[17] studied distinctly fed groupers, *E. coioides* from an Indonesian finfish mariculture farm for ecto- and endohelminth parasites. Pellet-fed *E. coioides* were infested with 13 parasite species/taxa of which six had a monoxenous (single host) and seven a heteroxenous (multi host) life cycle. A total of 14 parasite species/taxa were found in the fish that were fed with different trash fish species, four of them with a monoxenous and ten with a heteroxenous life cycle. The use of pellet food significantly reduced the transfer of endohelminths and the number of parasites with a heteroxenous life cycle. The risk of parasite transfer can be also reduced by feeding selected trash fish species with a lower parasite burden, using only trash fish musculature or minimizing the abundance of invertebrates (fouling) on the net cages. For *E. fuscoguttatus* Rueckert *et al*.[11] recorded a parasite infracommunity ranging from one to nine (cultured) and three to 14 parasite species (wild) also in Lampung Bay. In the present study, *E. fuscoguttatus* from the net cages had less parasites than those caught in the surrounding reef, however, at a low level.

The highest Shannon-Wiener diversity (total biodiversity) was revealed for *A. mate* from Jakarta Bay (1.4), followed by *E. sexfasciatus* from Cilacap (1.3). With respect to endoparasite diversity, we observe a trend with usually higher endoparasite diversity in the free-living epinephelids vs. the cultured *E. fuscoguttatus*, and for fish from Cilacap vs. fish from Jakarta Bay. This is also reflected by the Ec/En ratio from *E. fuscoguttatus* from cultured (1.5) compared with free-living (0.8) fish. The endoparasite diversity is of importance because under natural environmental conditions, the endoparasite richness inside the gut is regularly high and is used as bioindicator [60, 61]. Under polluted and heavily impacted environmental conditions, endoparasites lack the ability to complete their life cycles, and cannot be found in the studied fish. Consequently, our observation follows the general assumptions that the number of endoparasite species is low inside the mariculture fish as well as from polluted waters such as Jakarta Bay. The only exception here is *A. mate* that had similar high endoparasite diversity in Jakarta Bay and Cilacap. However, this might be caused by the small number of analyzed fish, or the migratory behavior that is known for this pelagic species.

According to our data, the number of fish parasites of wild fish exceeds that of mariculture fish [11, 42]. This coincided with the observation that tropical wild fish show fewer signs of diseases, though potential pathogens can be regularly found in the environment. In contrast, bacterial disease outbreaks occur under aquaculture conditions where only few parasites occur. While the diet (in our case the use of trash fish) and/or the environment can influence the number of revealed endoparasites in the fish, the parasite infracommunity as well might influence the microbiome, and suppress the impact of pathogenic bacteria and subsequently disease outbreaks. Consequently, we would expect to observe differences in the microbiome of the sampled parasitized or less infected fish.

The results of the microbial communities enable the identification of three potentially pathogenic bacteria, i.e. *Vibrio* sp., *Flavobacterium* sp. and *Photobacterium* sp‥ Comparing these results with the recorded parasite numbers using a Spearman’s rank-order correlation test, shows a weak negative correlation for *Vibrio* sp. and *Photobacterium* sp. In case of *Flavobacterium* sp. a weak positive correlation could be detected. The highest number of parasites were observed for free-living *E. sexfasciatus* from Cilacap, followed by *A. mate* and *E. sexfasciatus* from Jakarta Bay, and free-living and mariculture *E. fuscoguttatus* from Thousand Islands. All highly parasitized fish (above 50 individual metazoans) had no potentially pathogenic *Vibrio* sp., *Flavobacterium* sp. or *Photobacterium* sp. Instead, highest *Vibrio* sp. counts were only found in *E. fuscoguttatus* from inside the net cages and, to a much lower degree, in *E. fuscoguttatus* outside the net cages from surrounding reef. *Flavobacterium* sp. was only recorded from a fish inside the net cages without metazoan parasites, and *Photobacterium* sp. was recorded only from *E. fuscoguttatus*, from free-living and mariculture fish from Thousand Islands, without any record from the other two sampled fish species from Jakarta Bay and off Cilacap. This coincides with our assumption that there is a positive influence of the metazoan parasite infection on fish health and the occurrence of potential pathogenic bacteria inside the fish. However, this requires verification in future studies with a larger sample size.

## Conclusions

Notably the core microbiomes of both phylogenetically related and distant related fish species, *Epinephelus fuscoguttatus*, *Epinephelus sexfasciatus* and *Atule mate*, contained approximately the same classes of bacteria independent on the degree of pollution. However, the proportions of these bacterial classes strongly varied. The microbial biodiversity of two phylogenetically distant fish species, *A. mate* and *E. sexfasciatus* from Jakarta Bay and Cilacap were more closely related than those of the two phylogenetically adjacent species, *E. fuscoguttatus* and, *E. sexfasciatus* from Jakarta Bay, Cilacap and Thousand Islands. In addition, we detected weak negative correlation between the load of selected bacterial pathogens, *Vibrio* sp., *Photobacterium* sp. and the number of endoparasites. In the case of *Flavobacterium* sp. we found the opposite weak positive correlation. Of the three pathogenic bacterial genera, *Vibrio* sp. were found predominantly in *E. fuscoguttatus* from mariculture, and fewer in the vicinity of the net cages and rarely in fish from the heavily polluted waters from Jakarta Bay. *Flavobacterium* sp. showed highest counts inside maricultured fish and *Photobacteria* sp. was most prominent inside and close to the net cages. Due to our sample size, further study is required to make general statements concerning these findings, which are highly relevant for future finfish mariculture activities and management practices.

## Supporting Information

S1 Fig Number of raw sequence reads, merged paired reads, post-QA/QC sequence reads, and number of taxonomically classified reads. The light grey and dark grey dashed lines represent the average number of raw sequence reads and taxonomically classified sequence reads across all samples.
(TIFF)

S2 Fig Rarefaction curves. (TIFF)
