# Supplementary figures and images for: Microbial Diversity and Parasitic Load in Tropical Fish of Different Environmental Conditions
Source: PLoS One. 2016 Mar 28;11(3):e0151594. doi: 10.1371/journal.pone.0151594 (PMC4809571; doi:10.1371/journal.pone.0151594)

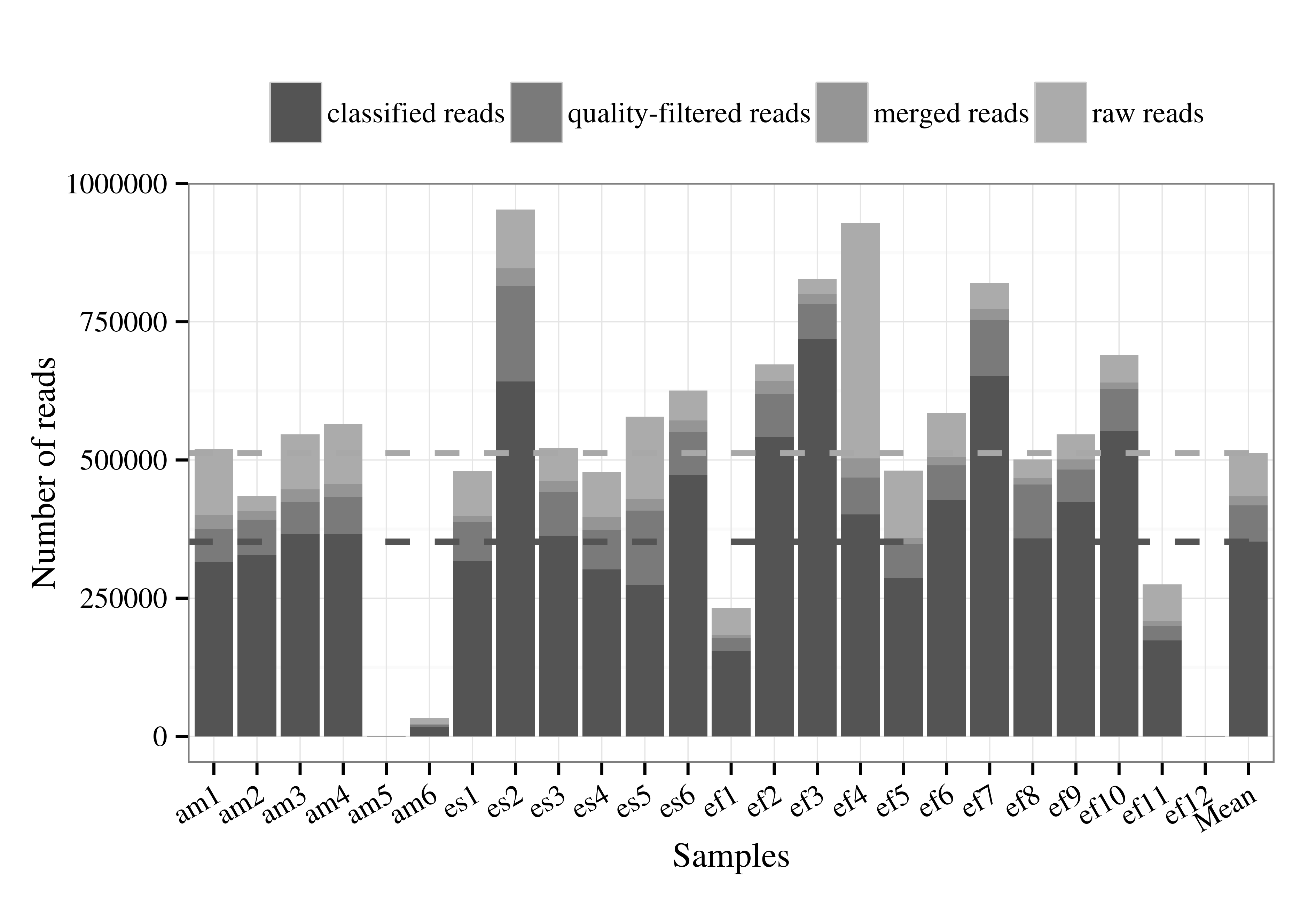

Supplement: S1 Fig — The light grey and dark grey dashed lines represent the average number of raw sequence reads and taxonomically classified sequence reads across all samples. (TIFF) [file pone.0151594.s001.tiff]

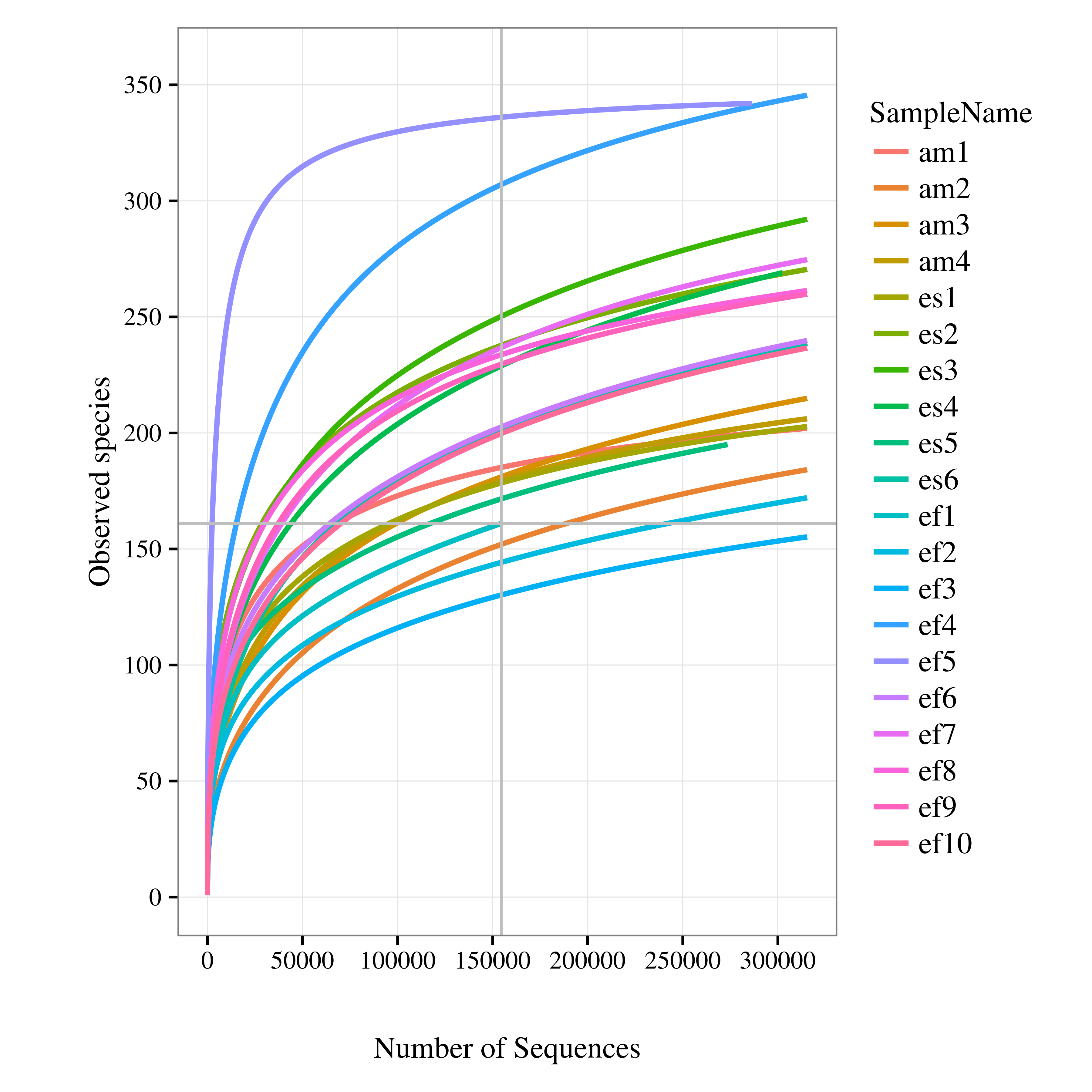

Supplement: S2 Fig — (TIFF) [file pone.0151594.s002.tiff]
